# Repeat Expansions with Small TTTCA Insertions in *MARCHF6* Cause Familial Myoclonus without Epilepsy

**DOI:** 10.1002/mds.30192

**Published:** 2025-04-09

**Authors:** Theresa Kühnel, Elsa Leitão, Renate Lunzer, Fabian Kilpert, Sabine Kaya, Claudia Del Gamba, Kelly Astudillo, Steven Frucht, Marion Simonetta‐Moreau, Eric Bieth, Iris Unterberger, Giulietta Maria Riboldi, Christel Depienne

**Affiliations:** ^1^ Institute of Human Genetics University Hospital Essen, University Duisburg‐Essen Essen Germany; ^2^ Institut für Humangenetik Medizinische Universität Innsbruck Innsbruck Austria; ^3^ Department of Neurology, The Marlene and Paolo Fresco Institute for Parkinson's and Movement Disorders NYU Langone Health New York New York USA; ^4^ Department of Neurology ASL Toscana Centro Prato Italy; ^5^ Department of Neurosciences University Hospital of Toulouse France, NeuroImaging Center, INSERM, UMR1214 Toulouse France, Paul Sabatier Toulouse 3 University Toulouse France; ^6^ Génétique Médicale Centre Hospitalier Universitaire de Toulouse Toulouse France; ^7^ Department of Neurology Innsbruck Medical University Innsbruck Austria

**Keywords:** myoclonus, repeat expansion, epilepsy, *MARCHF6*, familial adult myoclonus epilepsy (FAME)

## Abstract

**Background:**

Familial adult myoclonus epilepsy (FAME) is a rare autosomal dominant disorder caused by the same intronic TTTTA/TTTCA repeat expansion in seven distinct genes. TTTTA‐only expansions are benign, whereas those containing TTTCA insertions are pathogenic.

**Objective:**

We investigated the genetic basis of dominant cortical myoclonus without seizures in two unrelated families.

**Methods:**

Repeat‐primed polymerase chain reaction (PCR), long‐range PCR, and nanopore sequencing were used to detect and characterize expansions at known FAME loci.

**Results:**

We identified a novel repeat expansion in *MARCHF6*, comprising 388 to 454 elongated TTTTA repeats and 5 to 11 TTTCA repeats at the 3′‐terminus, segregating with cortical myoclonus in 8 affected individuals. This configuration shows meiotic stability but low‐level somatic variability in blood. We observed an inverse correlation between the number of TTTCA repeats and the age at myoclonus onset.

**Conclusions:**

These findings indicate that as little as five TTTCA repeats combined with expanded TTTTA repeats can cause cortical myoclonus without epilepsy, highlighting the potential mechanisms underlying FAME pathophysiology. © 2025 The Author(s). *Movement Disorders* published by Wiley Periodicals LLC on behalf of International Parkinson and Movement Disorder Society.

Familial adult myoclonus epilepsy (FAME), also known as familial adult myoclonic epilepsy, benign adult familial myoclonic epilepsy (BAFME), autosomal dominant cortical myoclonus and epilepsy (ADCME), or familial cortical myoclonic tremor with epilepsy (FCMTE),^1^, ^2^, ^3^ is a rare autosomal dominant disorder characterized by cortical myoclonus and epilepsy. Patients present with brief, irregular, involuntary movements, mainly in the upper limbs and occasionally in the lower limbs. Cortical tremor, the rhythmic variant of cortical myoclonus, may be present in some patients. Generalized tonic–clonic or myoclonic seizures are present in 15% to 100% of cases.^4^ Symptoms typically start in adulthood about the third decade, but the age of onset may vary from 10 to >60 years. The progression of the disorder also varies among individuals, with some experiencing mild symptoms that remain stable over time, whereas others may develop more severe symptoms that worsen with age.^1^, ^2^, ^3^

FAME is caused by the same pentanucleotide repeat expansion in introns of seven different genes: *SAMD12* (FAME1/BAFME1), *STARD7* (FAME2/FCMTE2/ADCME), *MARCHF6* (FAME3/FCMTE3), *YEATS2* (FAME4/BAFME4), *TNRC6A* (FAME6/BAFME6), *RAPGEF2* (FAME7/BAFME7), and *RAI1* (FAME8/BAFME8).^5^ Pathogenic expansions consist of elongated TTTTA repeats always containing a TTTCA insertion absent in healthy populations. These dynamic expansions, ranging from 2.2 to 18.4 kb, exhibit somatic variability. The typical structure is 5′‐(TTTTA)exp(TTTCA)exp‐3′, but TTTCA repeats can also insert within TTTTA repeats^6^, ^7^ or combine with other pentanucleotide motifs.^7^, ^8^ TTTTA‐only expansions, existing in some populations (eg, 6% of Japanese in *SAMD12*), are benign.^6^

FAME repeat expansions exhibit geographic clustering and population‐specific prevalence due to founder effects. FAME1 (*SAMD12*) predominantly occurs in Asian populations (Japan, China, India, and Sri Lanka),^9^ whereas FAME2 (*STARD7*) and FAME3 (*MARCHF6*) are mainly found in European patients, with FAME2 prevalent near Naples, Italy.^10^, ^11^, ^12^ Rare subtypes include FAME4 (Thailand), FAME6 and FAME7 (Japan), and FAME8 (Mali), potentially linked to specific geographic backgrounds.^6^, ^13^, ^14^ FAME subtypes may exhibit slightly distinct clinical characteristics. For instance, more than 70% of FAME3 patients exhibit seizures, sometimes as the first symptom. FAME2 and FAME3 cases occasionally present with focal rather than generalized seizures.^11^, ^15^, ^16^, ^17^

In this study, we investigated two unrelated families with cortical myoclonus but no seizures in which we identified a new repeat expansion configuration at the *MARCHF6* FAME locus.

## Patients and Methods

### Patients

**FIG. 1:**
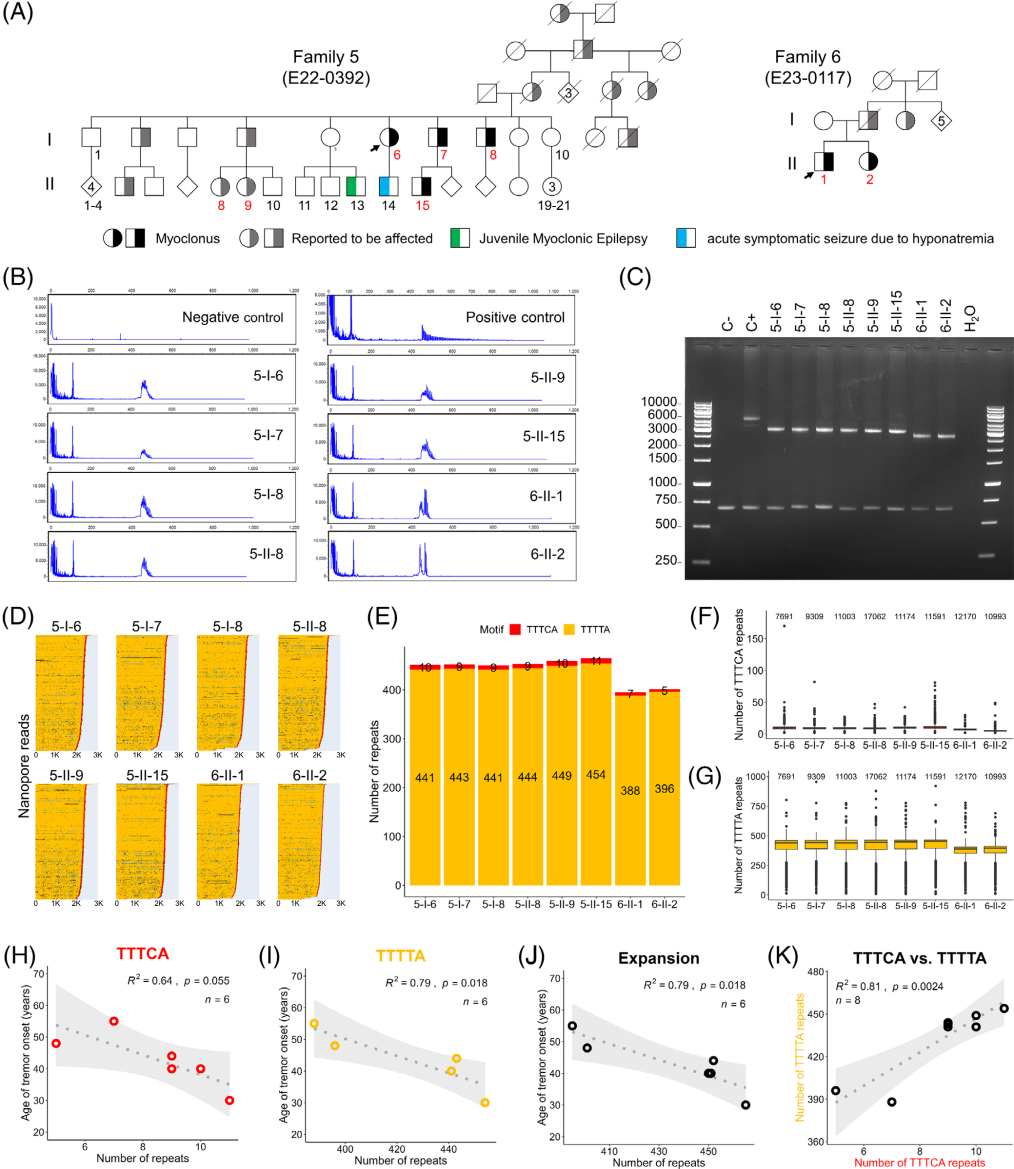
(**A**) Pedigrees of families 5 and 6. Half‐black symbols indicate affected individuals with neurological examination. Squares indicate males, circles indicate females, and diamonds indicate both sexes. Half‐gray symbols indicate affected subjects with a history but no examination. Half‐green symbol: juvenile myoclonic epilepsy; Half‐blue symbols acute symptomatic seizure due to hyponatremia. The numbers in the symbols indicate the number of siblings of the same sex. The numbers below the symbols correspond to the individual ID within the generation (left side of the pedigree). Numbers in red indicate an expansion, and numbers in black indicate its absence. (**B**) Identification of the expansion at the *MARCHF6* locus using repeat‐primed polymerase chain reaction (RP‐PCR). RP‐PCR profiles using TTTCA primer for a control individual (top left, negative control), a patient with a typical expansion in *MARCHF6* (top right, positive control), and the 8 affected individuals from families 5 and 6. (**C**) Gel electrophoresis of long‐range PCR (LR‐PCR) amplicons spanning the *MARCHF6* expansion in individuals with myoclonus from families 5 and 6. (**D**–**G**) Characterization of repeat expansion sequence and variability using nanopore sequencing. (**D**) Waterfall plots showing selected nanopore reads after separation of alleles based on the presence of at least two consecutive TTTCA repeats (Methods). Three hundred randomly chosen reads are shown in each graph. Yellow: TTTTA, red: TTTCA, cyan: TTTTG, blue: TTATG, black: other. (**E**) Median number of TTTTA and TTTCA repeats calculated from nanopore reads for each of the 8 affected individuals from families 5 and 6. (**F**) Box plots showing the distribution of the number of TTTCA repeats in each affected individual. (**G**) Box plots showing the distribution of the number of TTTTA repeats in each affected individual. (**H**–**J**) Genotype–phenotype correlations. Pearson's correlations of the age at onset (AAO) and the median number of (**H**) TTTCA repeats, (**I**) TTTTA repeats, and (**J**) median size of the expansion including both motifs. (**K**) Pearson's correlation between the median number of TTTCA and TTTTA repeats.

Two unrelated families of European descent, with multiple individuals affected with autosomal dominant cortical myoclonus (Fig. 1A), were referred for genetic testing of FAME expansions in a research context. Family 5 (E22‐0392) is of Austrian origin and includes 9 individuals with cortical myoclonus, 6 of whom were sampled. Family members were evaluated at the Institute of Human Genetics and the outpatient seizure clinic of the Department of Neurology, Innsbruck Medical University. Family 6 (E23‐0117) is of French origin and includes 4 affected members, 2 of whom were available for genetic analysis. Family members were evaluated at the Fresco Institute for Parkinson's Disease, NYU Langone Health NY, USA, and the University Hospital Toulouse, France. Genetic testing was conducted at the Institute of Human Genetics, University Hospital Essen. Informed consent was obtained for all family members. The study was approved by ethics boards at NYU Langone Health (s21‐00207) and the University Hospital Essen (18‐8176‐BO, 21‐10155‐BO).

### Expansion Detection and Characterization

Repeat‐primed polymerase chain reaction (RP‐PCR) was performed as previously described.^11^ Repeat expansions at the *MARCHF6* locus were amplified using long‐range PCR (LR‐PCR) from genomic DNA extracted from blood. Samples were barcoded, multiplexed, and sequenced using nanopore sequencing on MinION Mk1B R9.4.1 flow cells (Data S1). Basecalling and analysis of nanopore data were performed using Guppy,^18^ pycoQC,^19^ NanoPlot^20^ and BBMap^21^. Flanking sequences were trimmed off using Cutadapt.^22^ This in‐house bioinformatics pipeline using Snakemake^23^ is available on Github (https://github.com/kilpert/FAME3_analyses.git).

## Results

Clinical data were available for 4 affected members of family 5. At neurological examination, symptoms included cortical myoclonus in the lower more than the upper limbs, mild dysdiadochokinesia, ataxia, or action tremor (Table 1). These symptoms remained relatively stable over time. Routine electroencephalography images showed interictal generalized spike‐and‐wave discharges, but none had epilepsy. Two family members without myoclonus experienced seizures: one due to hyponatremia and another due to juvenile myoclonic epilepsy.

**TABLE 1:** Summarized clinical features of examined family members of families 5 and 6

Family	5	5	5	5	6^a^	6	6
Individual	5‐I‐6	5‐I‐7	5‐I‐8	5‐II‐15	6‐I‐2	6‐II‐1	6‐II‐2
Sex	F	M	M	M	M	M	F
Age (y)	68	67	65	37	80 (deceased)	63	52
AAO (y)	40	44	40	27	50	55	48
Age at myoclonus/tremor onset (y)	40	44	40	30	50	55	48
Symptom at onset	Action/exercise‐induced myoclonus of the legs with sudden weakness and coordination problems, occasional falls	Action/exercise‐induced myoclonus of the legs with sudden weakness and coordination problems, occasional falls	Action/exercise‐induced myoclonus of the left leg and weakness	Sudden motor arrest—stiffening of the legs—not able to perform ongoing motor activity; repeatedly occurring during disease onset	N/A	Myoclonic jerks in the upper limbs mostly triggered by fine motor activities and by stress	Mild intermittent rest and action, upper‐ more than lower‐limb myoclonus triggered by stress and worsening during sleep
Cortical myoclonus (Y/N)	Y legs, arms—mild	Y legs, arms—mild	Y legs, arms—mild	Y arms, legs	Y arms, legs	Y	Y arms, legs
Cortical tremor (Y/N)	Y—mild	Y—mild	Y—mild	N	Y	N	N
Triggering factors	Motor activity overall, particularly walking on uneven ground, hiking in steep terrain	Motor activity overall, particularly walking on uneven ground, hiking in steep terrain, exercise (cycling)	Motor activity overall, particularly motor activity on uneven ground, hiking/skiing in steep terrain, exercise (cycling)	Motor activity arms (writing, carrying items); motor activity legs overall, particularly motor activity on uneven ground, exercise (cycling, soccer, tennis)	Stress and standing for a prolonged period of time	Stress and fine more tasks	Sleep, inactivity, and fine motor tasks
Worsening factors	Emotional stress, sleep deprivation, fever	Sleep disturbance, alcohol	Emotional stress, sleep deprivation, fever	Emotional stress, sleep deprivation	Stress	Stress	Stress and sleep
Treatment	VPA‐beneficial	VPA‐beneficial	VPA‐beneficial	LEV‐beneficial	TPM, LEV‐beneficial	None	LEV‐beneficial
Seizures (Y/N)	N	N	N	N	N	N	N
Cognitive abilities	Normal	Normal	Normal	Normal	N/A	Normal	Normal
Anxiety	Y	N/A	N/A	N/A	Y	Y	Y
OCD	N/A	N/A	N/A	N/A	N	Y	N/A
Other	N/A	Insomnia	Insomnia	Syncope	None	None	N/A
EEG	Generalized SPW	Generalized SPW	Generalized SPW	Generalized SPW	Generalized SPW	N/A	Normal
Cerebral MRI	Nonlesional	Nonlesional	Nonlesional	Nonlesional	Nonspecific cortical atrophy	Nonlesional	Olfactory meningioma treated by radiosurgery
Neurological exam	Resting tremor, action tremor, bilateral dysdiadochokinesis, ataxia	Action tremor, bilateral dysdiadochokinesis, ataxia	Action tremor	Mild bilateral dysdiadochokinesis	Upper‐ and lower‐limb myoclonus	Mild and occasional myoclonic jerks on the left more than the right upper limb, especially with fine motor skills (i.e., writing and drawing a spiral)	No tremor. No ataxia. Hand clumsiness in fine motor tasks possibly related to post radiotherapy corpus callosum lesion

^a^ Reported by the son.

Abbreviations: AAO, age at onset; N/A, not assessed or not available; Y, yes; N, no; VPA, valproic acid; LEV, levetiracetam; TPM, topiramate; OCD, obsessive‐compulsive disorder; EEG, electroencephalography; SPW, spike and waves; MRI, magnetic resonance imaging.

The proband of family 6 (6‐II‐1) was a 63‐year‐old man with an 8‐year history of action‐induced cortical myoclonus of the upper limbs triggered by stress, associated with anxiety and obsessive‐compulsive features. His father (deceased) and sister presented with similar but more severe symptoms (both myoclonus and anxiety) with progression over time. The sister presented generalized cortical myoclonus that worsened during sleep. There was no personal or family history of seizures.

Across the two identified families, the age at myoclonus onset ranged from 27 to 55 years. Treatment with antiseizure medications, including valproic acid, topiramate, and levetiracetam, was beneficial in all of the 5 treated subjects but did not entirely suppress myoclonus.

In addition to cortical myoclonus, 5 affected family members (4 in family 5 and the sister in family 6) reported brief paroxysmal episodes of ataxia and sudden leg weakness, triggered by motor activity or prolonged exercise such as walking on uneven ground or steep terrain, going up and down the stairs, or dancing. These episodes were not observed in consultation, and their nature could not be precisely determined, but their description may correspond to negative myoclonus.

We tested the presence of repeat expansions at the FAME2 (*STARD7*) and FAME3 (*MARCHF6*) loci using RP‐PCR. This analysis was negative for FAME2 but revealed a sawtooth pattern that stopped abruptly after a few peaks in contrast to the positive control, displaying the classical continuous pattern associated with a long TTTCA expansion at the FAME3 locus (Fig. 1B). We then amplified the alleles at this locus using LR‐PCR in all available members of both families. A PCR product between 2 and 3 kb was identified in the 2 available affected individuals of family 6 (E23‐0117) and all individuals with myoclonus in family 5 (E22‐0392), whereas the 15 family members without myoclonus did not have the expansion (Fig. 1C; Fig. S1). To investigate the repeat number, we sequenced the LR‐PCR amplicons of the 8 individuals with an expanded allele using nanopore sequencing. This analysis revealed a comparable expansion structure in the two families, with an expanded TTTTA fragment ranging from 385 to 453 repeats, followed by a short TTTCA stretch composed of 5 to 11 repeats ending with three TTTTA repeats in family 5 and one TTTTA repeat in family 6 (Fig. 1D,E). Interestingly, nanopore sequencing, which was done on a new flow cell and multiplexing only the samples included in this study, also detected reads with a higher number of TTTCA repeats in all individuals although this phenomenon was limited (Fig. 1F), as well as a somatic variability in TTTTA repeat numbers (Fig. 1G).

Finally, we investigated whether there was any correlation between the age at onset (AAO) and the number of repeats. We observed significant inverse correlations between the AAO and the number of both TTTTA and TTTCA repeats as well as the size of the expansion (Fig. 1H,I). In addition, we observe that the number of TTTCA repeats was positively correlated to the number of TTTTA repeats (Fig. 1K).

## Discussion

FAME represents a new paradigm of noncoding repeat expansion disorders in which the same intronic expansion is pathogenic regardless of the function of the gene harboring the expansion. The seven genes where FAME expansions occur encode proteins with completely different functions and subcellular localizations, and expansions do not appear to alter gene expression or splicing.^24^ The pathophysiological mechanisms leading to FAME remain largely unknown,^24^, ^25^ but currently favored hypotheses include the accumulation of expansion‐carrying RNA molecules in RNA foci followed by sequestration of RNA‐binding proteins as in myotonic dystrophy,^26^, ^27^, ^28^ or the expression of toxic polypeptides as a result of repeat‐associated non‐AUG (RAN) translation.^29^

Here, we describe two families with a new expansion in *MARCHF6*, associated with a milder phenotype characterized by different degrees of cortical myoclonus but no seizures. In addition, some family members reported action‐induced paroxysmal episodes. As no neurophysiology or videos were available, the nature of these movement disorders remains unclear. The expansion, consisting of expanded TTTTA repeats associated with 5–11 TTTCA repeats at its 3′‐extremity, segregates with cortical myoclonus in both families. The existence of short TTTCA stretches within *SAMD12* expansion has been reported in two previous studies: 1 isolated patient had an expansion containing 14 TTTCA repeats,^7^ whereas a small family with 2 affected individuals had a 4.5‐kb expansion containing 10 TTTCA repeats interrupted by 7 TTTTA repeats.^30^ In both cases, patients had late‐onset seizures. Our findings provide definite evidence of segregation of these atypical expansions with cortical myoclonus and further show that as little as five TTTCA repeats next to TTTTA expansions are sufficient to lead to cortical myoclonus. Although we observed low levels of somatic mosaicism with longer TTTCA stretches in nanopore data, these expansions remained relatively stable during transmission, consistent with the absence of anticipation in these families. Nevertheless, we observed an inverse correlation between the age at myoclonus onset and repeat number, which applies to both TTTTA and TTTCA repeats, as they correlate with each other. This suggests that introducing a “C” into an AT‐rich sequence may be selectively favored, potentially turning nonpathogenic TTTTA expansions into pathogenic repeats.

The observation that myoclonus occurs in the presence of a few TTTCA repeats, whereas seizures tend to occur with larger TTTCA expansions, suggests that these clinical features may result from different degrees of cortical hyperexcitability and/or distinct effects of the expansion in different brain regions (e.g., cerebellum and cortex). This aligns with our previous finding that the number of TTTCA repeats correlates with the age of seizure onset but not the onset of myoclonus.^11^ Moreover, due to the benign nature of expansions exclusively consisting of TTTTA repeats, the fact that a few TTTCA repeats alongside TTTTA expansion is sufficient to confer pathogenicity raises questions about current hypotheses. Although this does not entirely rule out the ability of the corresponding RNA to aggregate into RNA foci and sequester RNA‐binding proteins or to express toxic polypeptide by RAN translation, it is more likely that the introduction of TTTCA repeats may modify the ability of the DNA or the RNA to fold or to bind protein partners. TTTCA repeats adjacent to TTTTA are rare in the human reference genome, with one occurrence in the *MED12L* gene. The reason why expression of TTTCA adjacent to TTTTA repeats is nonpathogenic in the context of *MED12L* is of interest, but it is worth observing that the expression of this gene remains low in the brain (as well as in other tissues).

In conclusion, our results confirm the pathogenicity of expansions containing small TTTCA stretches and further contribute to a better understanding of FAME conditions. It also implies that FAME loci should be tested in patients who have cortical myoclonus even in the absence of a familial history of epilepsy.

## Author Roles

1. Research project: A. Conception, B. Organization, C. Execution; 2. Statistical analysis: A. Design, B. Execution, C. Review and critique; 3. Manuscript: A. Writing of the first draft, B. Review and critique.

T.K.:1A, 1B, 1C, 3B.

E.L.:1C, 2A, 2B, 3A, 3B.

R.L.:1B, 1C, 3B.

F.K.:1C, 2C, 3B.

S.K.:1B, 1C, 3B.

C.D.G.:1C, 3B.

K.A.:1C, 3B.

S.F.:1C, 3B.

M.S.M.:1C, 3B.

E.B.:1C, 3B.

I.U.:1C, 3B.

G.M.R.:1B, 1C, 3B.

C.D.:1A, 1B, 1C, 3A, 3B.

## Full financial disclosures of all authors for the previous 12 months

This work was supported by Deutsche Forschungsgemeinschaft (DFG) grants (project numbers 455314768 and 458099954) to C.D. G.M.R. was supported by grants from Michael J Fox Foundation, Parkinson’s Foundation, Department of Defense (PD210038), NIH (R01 NS116006; R01 NS133742; 1OT2OD038130). M.S.M. has received Honoraria from IPSEN BIOTECHfor training courses and ABBVIE for lectures. All remaining authors had no ﬁnancial disclosures or conflict of interest to report.

## Supporting information


**Data S1.** Supporting Information.

## Data Availability

Nanopore reads have been deposited in the European Genome‐Phenome Archive (EGA, http://www.ebi.ac.uk/ega) (https://ega-archive.org/studies/EGAS50000000570). All other data supporting the findings described in this manuscript are available in the article or can be requested from the corresponding author.
