# Supplementary material for: Repeat Expansions with Small TTTCA Insertions in MARCHF6 Cause Familial Myoclonus without Epilepsy
Source: Mov Disord. 2025 Apr 9;40(7):1401–8. doi: 10.1002/mds.30192 (PMC12273606; doi:10.1002/mds.30192)
Supplement: Supplementary file 1 — Data S1. Supporting Information. [file MDS-40-1401-s001.docx]

**Supplementary Information**

**Supplementary Methods**

***Patients***. Family 5 (E22-0392) originates from Austria and family members were seen at the Institute of human genetics, Medical University of Innsbruck, Austria, and are treated at the outpatient seizure clinic of the Department of Neurology, Innsbruck Medical University. Family 6 (E23-0117) is a family of French origin. The index case was seen at the Fresco Institute for Parkinson’s disease at NYU Langone Health NY, USA, while his sister was referred first to the medical Neurosciences Department, then to the Medical Genetics department of University Hospital Toulouse, France. Informed consent was obtained from patients in accordance with the Declaration of Helsinki. The study was approved by the Institutional Review Board of NYU Langone Health (Movement Disorder Genomic Study, s21-00207). Genetic testing was performed at the Institute of Human Genetics, University Hospital Essen, Germany. The study on Familial Adult Myoclonic Epilepsy (FAME) have received the approval of the ethics committee of University Hospital Essen (18-8176-BO). Inclusion of family 5/E22-0392 was part of the project “Identification of tandem repeat EXPAnsions in unsolved Neurological Disorders” (EXPAND), which has also received the approval of the ethics committee of University Hospital Essen (21-10155-BO).

***Repeat-primed PCR.*** Repeat-primed PCR (RP-PCR) was performed as previously described.^11^ Amplification was performed from 100 ng genomic DNA using 0.8 μM of the 6-FAM labeled FAME3-P1R (GGAAAAGGGAGGGTTATAGAGGA), 0.8 μM primer P3-PU (TACGCATCCCAGTTTGAGACG), and 0.08 μM primer P3-TTTCA (TACGCATCCCAGTTTGAGACG-TTCATTTCATTTCATTTCATTTC) using the HotStarTaq Master Mix (QIAGEN). PCR amplification cycles consisted in a first step of 95 °C for 15 min, followed by 40 cycles (94 °C for 1 min, 58 °C for 1 min, and 72 °C for 2 min 30 s) and a final extension step (72 °C for 10 min). RP-PCR products were detected on an ABI 3130xl DNA Analyzer and analyzed using GeneMapper® software v5.0 (Thermo Fisher Scientific).

***Long-range PCR amplification***. Repeat expansions at the *MARCHF6* locus were amplified by Long-Range PCR (LR-PCR) from genomic DNA extracted from blood using the PrimeSTAR GXL DNA Polymerase (Takara) and the following primers: Forward primer (FAME3_LR-PCR_F2): CACTTAAAGGAAAAGGGAGGGTTATAGAGGA; Reverse primer (FAME3_LR-PCR_R2): CGCACGGTTGATGTGTTTGTAACAT. PCR was performed with 100 ng genomic DNA, and 0.24 µM of each primer in 25 µl, using mix and dNTP conditions recommended by the manufacturer. The PCR program consisted in 26 cycles of the following steps: 98°C for 10 seconds, 60°C for 15 seconds and 68°C for 10 minutes. 75 µl of pooled LR-PCR reactions of each individual were purified using the DNA Clean & Concentrator (Zymo Research) before proceeding to DNA barcoding.

***Nanopore sequencing***. Samples were multiplexed and prepared for nanopore sequencing using the ligation-based sample preparation and native barcoding protocols (SQK-LSK109 with EXP-NBD196, Oxford Nanopore). This protocol includes the following steps: 1) End-prep (incubation of 200 fmol of each purified amplicon with NEBNext Ultra II End Repair/dA-tailing Module Reagents at 20°C for 5 min and 65°C for 5 min in a 96-well plate); 2) Native Barcoding ligation (incubation with native barcodes and NEB Blunt/TA Ligase Master Mix at 20°C for 20 min and 65°C for 10 min); 3) Pooling of barcoded amplicons and purification using AMPure XP beads (Beckman Coulter); 4) Adapter ligation (10-min incubation with Adapter Mix II Expansion / NEBNext Quick Ligation Reaction Module followed by clean-up with AMPure XP beads). We performed all steps according to the manufacturer’s recommendations. We loaded ~15 ng of the final prepared library onto a MinION Mk1B R9.4.1 flow cell. Nanopore sequencing was undertaken for 24-48 hours and monitored using the MinKNOW software. Basecalling and primary analysis of nanopore data were performed using Guppy^18^ (version 6.5.7) for basecalling, pycoQC^19^ (version 2.5.2) and NanoPlot^20^ (version 1.41.6) for quality control and to generate sequence_summary.txt, final_summary.txt, and fastq.gz files from fast5 files. Basecalling was performed with the “sup” model (dna_r9.4.1_450bps_sup.cfg) using the following parameters: ““--recursive --compress_fastq --do_read_splitting --calib_detect --records_per_fastq 0 --enable_trim_barcodes”. Fastq files were quality trimmed and filtered using BBMap^21^ bbduk.sh with the parameters: “-Xmx2g qin=33 minlen=200 qtrim=lr trimq=10 maq=10 maxlen=100000”. The analysis of the repeats was performed by a Snakemake^22^workflow (https://github.com/kilpert/FAME3_analyses.git) on the command line. We used the following flanking sequences: ACACTTATACTGTTGTGGTTCTGTT (chr5:10356314-10356338) upstream and CCGAGATGGAGTCTTGCCCTGTTGC (chr5:10356412-10356436) downstream of the repeat expansion to filter for reads that contained both sequences, allowing 2 mismatches per flanking sequence using bbduk.sh with --literal and --edist parameters. Reads where both flanking sequences showed a -strand orientation were subsequently converted to their reverse-complement (+strand) sequence. The reads were then filtered into two groups of reads: reads that contained two consecutive TTTCA repeats (i.e. TTTCATTTCA) and those without. Both groups were investigated further in the same way, with a particular interest on the TTTCATTTCA containing group. Flanking sequences were trimmed off from both sides using Cutadapt^23^ to isolate the repeat region. In addition, the workflow calculates statistics and generates plots (custom Python and R scripts) to characterize the nature of the repeat expansion in length and motif composition. Specific alleles (length and motif) can be defined manually for enhanced visualization.

**Supplementary Figure**


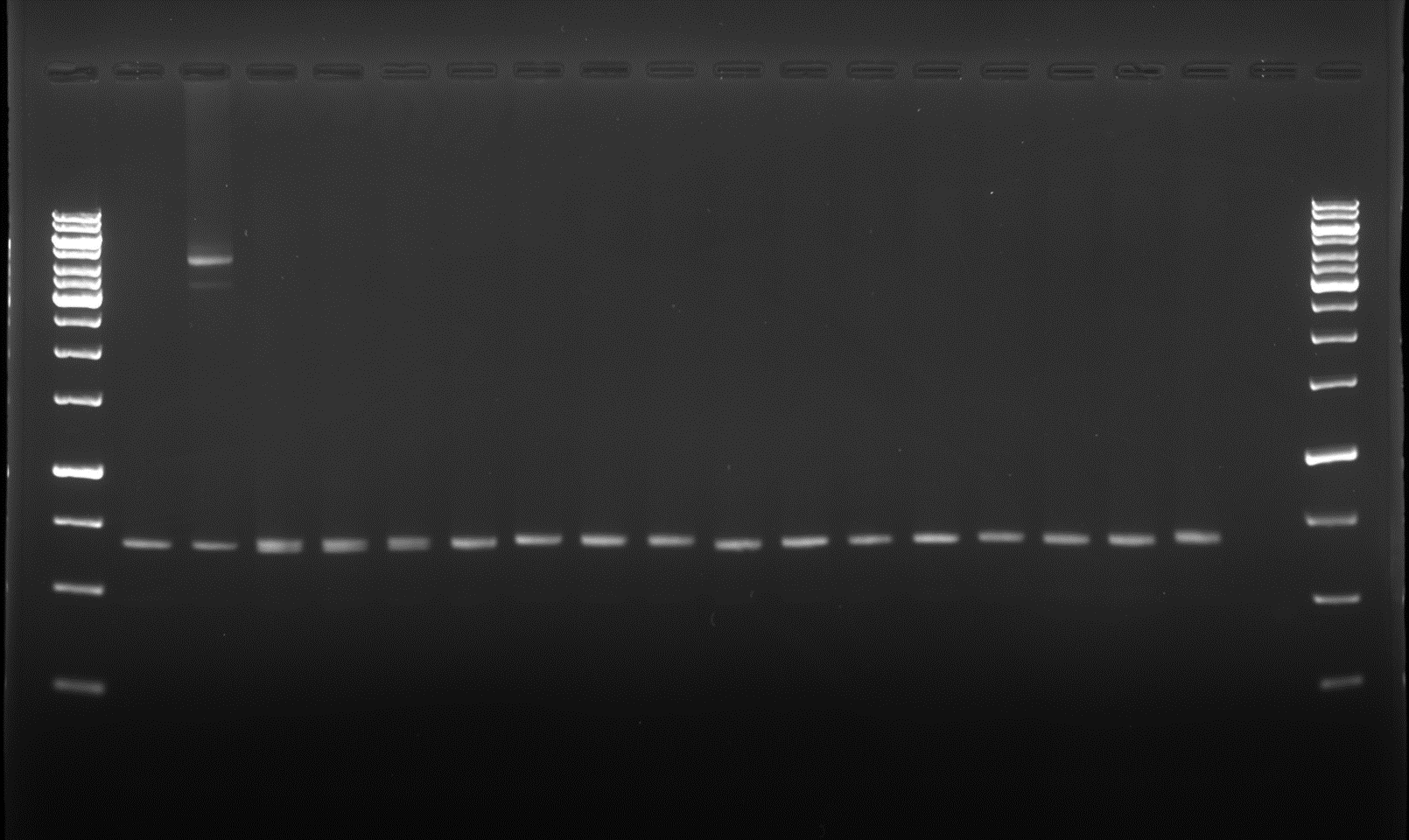


C-

C+

5-I-1

5-I-5

5-I-10

5-II-1

5-II-2

5-II-3

5-II-4

5-II-10

5-II-11

5-II-12

5-II-13

5-II-14

5-II-19

5-II-20

5-II-21

H_2_O

250

500

750

1000

1500

2000

3000

6000

10000

**Supplementary Figure 1.** Gel electrophoresis of LR-PCR amplicons showing the presence of normal alleles only on other members from both families.
